# Minimalistic approach to enhanced recovery after pediatric scoliosis surgery

**DOI:** 10.1007/s43390-023-00675-0

**Published:** 2023-03-19

**Authors:** Scott A. Barnett, Bryant M. Song, Matthew Bauer, Matthew E. Nungesser, Claudia Leonardi, Michael J. Heffernan

**Affiliations:** 1 Children’s Hospital New Orleans, LSU Health Sciences Center, New Orleans, LA USA; 2 Louisiana State University Health Sciences Center, New Orleans, LA USA; 3 School of Public Health, LSU Health Sciences Center, New Orleans, LA USA; 4 Children’s Hospital Los Angeles, Keck School of Medicine of USC, Los Angeles, CA USA; 5 Jackie and Gene Autry Orthopaedic Center, Children’s Hospital Los Angeles, 4650 Sunset Blvd, Mailstop #69, Los Angeles, CA 90027 USA

**Keywords:** Adolescent idiopathic scoliosis, Enhanced recovery, Healthcare delivery

## Abstract

**Purpose:**

Prior studies of enhanced recovery protocols (ERP) have been conducted at large institutions with abundant resources. These results may not apply at institutions with less resources directed to quality improvement efforts. The purpose of this study was to assess the value of a minimalistic enhanced recovery protocol in reducing length of stay (LOS) following PSF for adolescent idiopathic scoliosis. We hypothesized that accelerated transition to oral pain medications and mobilization alone could shorten hospital length of stay in the absence of a formal multimodal pain regimen.

**Methods:**

AIS patients aged 10–18 who underwent PSF at a tertiary pediatric hospital between January 1, 2014 and December 31, 2017 were reviewed. The study population was further narrowed to consecutive patients from a single surgeon’s practice that piloted the modified ERP. Reservation from key stakeholders regarding the feasibility of implementing widespread protocol change led to the minimal alterations made to the postoperative protocol following PSF. Patients were divided into either the Standard Recovery Protocol (SRP) or Enhanced Recovery Protocol (ERP). Primary variables analyzed were hospital LOS, complications, readmissions, and total narcotic requirement.

**Results:**

A total of 92 patients met inclusion criteria. SRP and ERP groups consisted of 44 (47.8%) and 48 (52.2%) patients. There was no difference between the two groups with regard to age, sex, and ASA score (*p* > 0.05). Fusion levels and EBL did not differ between treatment groups (*p* > 0.05). PCA pumps were discontinued later in the SRP group (39.5 ± 4.3 h) compared to the ERP group (17.4 ± 4.1 h, *p* < 0.0001). Narcotic requirement was similar between groups (*p* = 0.94) Patients in the SRP group had longer hospital stays than patients in the ERP group (*p* < 0.0001). 83% of the ERP group had LOS ≤ 3 days compared to 0% in the SRP group, whose mean LOS was 4.2 days. There was no difference in complications between the groups (2.2% vs 6.0%, *p* = 0.62). Readmission to the hospital within 30 days of surgery was rare in either group (2 SRP patients: 1 superior mesenteric artery syndrome, 1 bowel obstruction vs 0 ERP patients, *p* = 0.23).

**Conclusion:**

In this cohort, minor changes to the postoperative protocol following surgery for AIS led to a significant decrease in hospital length of stay. This minimalistic approach may ease implementation of an ERP in the setting of stakeholder apprehension.

## Introduction

Enhanced recovery protocols (ERPs) accelerate postoperative recovery and improve hospital efficiency without increased complications or readmission rates [1–4]. Previous studies in adolescent idiopathic scoliosis (AIS) demonstrate decreased pain scores, earlier mobilization, and reduced hospital length of stay (LOS) with implementation of these protocols [5–7]. Key components of ERPs include preoperative patient counseling, perioperative pain management, and early patient mobilization [8]. Prior studies evaluating the efficacy of ERPs have been conducted at large institutions with abundant resources. These results may not be applicable at all institutions including institutions that lack a formalized quality improvement team and those with less experience implementing quality improvement efforts. Furthermore, postoperative protocols were overhauled in prior studies, including substantial changes to the postoperative pain medication regimen. In our context, there was reservation from key stakeholders (anesthesia, surgeons, and nursing) regarding the feasibility of implementing a widespread protocol change. Therefore, we initially obtained stakeholder consent to make minimal alterations to the postoperative protocol following posterior spinal fusion (PSF) in a single surgeon’s practice. Only two adjustments were made to the postoperative protocol including the timing of mobilization and the timing of transition to oral pain medications, while all other protocol components remained unchanged.

The purpose of this study was to assess the value of a modified, minimalistic enhanced recovery protocol in reducing LOS following PSF for adolescent idiopathic scoliosis. We hypothesized that accelerated transition to oral pain medications and mobilization alone could shorten hospital length of stay in the absence of a formal multimodal pain regimen.

## Methods

### Study design

We conducted an IRB-approved retrospective review of AIS patients aged 10–18 years who had undergone PSF at a single tertiary pediatric hospital between January 1, 2014 and December 31, 2017. Exclusion criteria included a non-idiopathic diagnosis (neuromuscular, congenital, syndromic), younger than 10 years at time of presentation, history of previous spinal surgery, or required multiple/staged surgeries. Patients previously treated with a brace were not excluded from this study. Due to stakeholder reservation, the study population was further narrowed to consecutive patients from a single surgeon’s practice that piloted the modified ERP.

### Treatment

Consecutive patients were included in the study. Patients treated from January 1, 2014 to April 30, 2016 formed the SRP group while those treated from May 1, 2016 through December 31, 2017 formed the ERP group. The SRP group received a weight dosed morphine patient-controlled analgesia (PCA) following surgery that was discontinued by noon on post-operative day (POD) 2. At this point, the patients were transitioned to an oral pain regimen consisting of hydrocodone 5 mg every 6 h, diazepam 2 or 5 mg every 6 h (dependent on patient age and weight), and intravenous morphine for breakthrough pain. SRP patients mobilized with physical therapy on POD 2. The ERP group also received a PCA following surgery; however, this was discontinued in the morning of POD 1. The oral pain regimen was identical between the ERP and SRP treatment groups. ERP patients were encouraged to mobilize on POD 1 with physical therapy. The preoperative clinic visit was pivotal for patient and family education. During this visit, the surgical plan and perioperative expectations were reviewed in depth. For the ERP, this included discussion of timing of transition to oral pain medications as well as early mobilization. All postoperative scoliosis patients were cared for on a single nursing unit in the hospital. Nurses and physical therapists on this unit were educated on the SRP and ERP so that there was no confusion among treatment teams.

### Variables and outcome measures

Demographic information and clinical characteristics including age, sex, American Society of Anesthesiologists (ASA) physical status classification, and curve magnitude were obtained from the electronic medical record. Surgical characteristics including the number of fused levels and estimated blood loss (EBL) were included. Postoperative outcomes analyzed were curve magnitude as well as the time from surgery until discontinuation of the patient-controlled anesthesia (PCA) pump and Foley removal. The primary outcomes were hospital LOS and total narcotic requirement. Perioperative complications and 30-day hospital readmissions were also documented.

### Statistical analysis

Data were analyzed using the SAS/STAT software version 9.4 of the SAS System for PC, © 2014 SAS Institute Inc., Cary, NC, USA. Demographic, baseline characteristics, and study outcomes were compared between the two groups using either χ^2^ tests or Fisher exact tests for categorical variables or Student *t*-test for continuous variables. Postoperative curve magnitude was analyzed including preoperative curve magnitude as covariates in a multivariable model. Statistical significance was defined as *p* value < 0.05.

## Results

A total of 92 consecutive patients met inclusion criteria and were managed with either the SRP or ERP treatment protocols. The SRP and ERP groups consisted of 44 (47.8%) and 48 (52.2%) patients, respectively. Patient demographics and preoperative clinical characteristics are summarized in Table 1. Most patients in the study cohort were female (77.3% SRP vs 70.8% ERP; *p* = 0.482). There was no difference between the two groups with regard to age, sex, or ASA score (*p* > 0.05).

**Table 1:** Patient demographics and preoperative clinical characteristics (*n* = 92)

Characteristics	SRP (*N* = 44)	ERP (*N* = 48)	*p*-value
Sex, [% (*n*)]			0.482
Female	77.3 (34)	70.8 (34)	
Male	22.7 (10)	29.2 (14)	
ASA physical status classification, [% (*n*)]			0.545
1	45.4 (20)	54.2 (26)	
2	52.3 (23)	41.7 (20)	
3	2.3 (1)	4.2 (2)	
Age (years), [mean (SD)]	14.4 (2.1)	13.8 (1.7)	0.137
Curve magnitude (°), [mean (SD)]	50.6 (11.2)	56.4 (14.1)	0.032

*ASA* American society of Anesthesiologists; *ERP* enhanced recovery protocol; *SD* standard deviation; *SRP* standard recovery protocol

Perioperative characteristics between SRP and ERP treatment groups are outlined in Table 2. There was no difference in the number of spinal levels fused and EBL between treatment groups (*p* > 0.05). The residual curve magnitude following surgery was lower in the SRP group (5.5° ± 0.6°) compared to the ERP group (8.3° ± 0.6°, *p* = 0.001). PCA pumps were discontinued later in the SRP group (39.5 ± 4.3 h) compared to the ERP group (17.4 ± 4.1 h, *p* < 0.0001). Similarly, Foley catheters were discontinued later in the SRP group (56.4 ± 10.0 h) compared to the ERP group (41.0 ± 10.3 h, *p* < 0.0001). Patients in the SRP group had longer hospital stays than patients in the ERP group (*p* < 0.0001). Eighty-three percent of the ERP group had LOS ≤ 3 days compared to 0% in the SRP group, whose mean LOS was 4.2 days.

**Table 2:** Perioperative characteristics for SRP and ERP treatment groups (*n* = 92)

Characteristics	SRP (*N* = 44)	ERP (*N* = 48)	*p*-value
Surgery characteristics			
Number of levels fused, [mean (SD)]	11.5 (1.9)	11.9 (1.8)	0.333
EBL (mL), [mean (SD)]	401 (152)	456 (220)	0.170
Post-surgery characteristics			
Postop curve magnitude (°), [LSM (SEM)]^a^	5.5 (0.6)	8.3 (0.6)	0.001
Time from surgery to PCA (h), [mean (SD)]	39.5 (4.3)	17.4 (4.1)	< 0.0001
Time from surgery to Foley removal (h), [mean (SD)]^b^	56.4 (10.0)	41.0 (10.3)	< 0.0001
LOS (days), [% (*n*)]			< 0.0001
1	0 (0)	0 (0)	
2	0 (0)	2.1 (1)	
3	0 (0)	81.2 (39)	
4	81.8 (36)	12.5 (6)	
5 +	18.2 (8)	4.2 (2)	

*EBL* estimated blood loss; *ERP* enhanced recovery protocol; *LSM* least square mean; *PCA* patient-controlled analgesia; *SEM* standard error of the mean; *SRP* standard recovery protocol

^a^Model included pre surgery values as covariate

^b^Exact time was not collected on all patients (*n* = 79)

Adverse events and narcotic requirements between treatment groups are presented in Table 3. There was no difference in complications between the SRP and the ERP groups (2.2% vs 6.0%, *p* = 0.618). Although not statistically significant, more patients in the SRP group were readmitted to the hospital within 30 days of surgery (*p* = 0.227). There was no difference in total narcotic medications administered between the two groups (*p* = 0.936).

**Table 3:** Adverse events and narcotic requirements for SRP and ERP treatment groups

Characteristics	SRP (*N* = 44)	ERP (*N* = 48)	*p*-value
Complications, [% (*n*)]	2.2 (1)	6.0 (3)	0.618
Readmission within 30 days, [% (*n*)]	4.4 (2)	0 (0)	0.227
In hospital total MME, [LSM (SEM)]	78.8 (4.9)	78.3 (4.7)	0.936
MME for patients in hospital, [LSM (SEM)]			
Day 1 (*n* = 96)	21.2 (1.4)	23.9 (1.3)	0.148
Day 2 (*n* = 95)	30.3 (1.7)	30.6 (1.6)	0.914
Day 3 (*n* = 55)	22.3 (1.8)	29.2 (4.0)	0.125
Day 4 (*n* = 11)	12.8 (5.0)	27.5 (10.6)	0.240

*ERP* enhanced recovery protocol; *LSM* least square mean; *MME* morphine milligram equivalent; *SEM* standard error of the mean; *SRP* standard recovery protocol

^a^Model included pre surgery values as covariate

## Discussion

An emphasis on efficiency and safety in healthcare delivery has generated a focus on ERPs in many surgical disciplines including pediatric scoliosis surgery [4, 5, 9]. The goals of ERPs are to accelerate postoperative recovery while reducing hospital LOS. Reducing LOS has many potential benefits including reduced healthcare cost, decreased familial socioeconomic burden, and a decreased risk of hospital-acquired infections [10–12]. These multi-faceted treatment protocols are typically developed and implemented by multidisciplinary teams and can include preoperative exercise, perioperative patient counseling, peer support groups, early patient mobilization, and anesthesia involvement with a revised perioperative pain management regimen (multimodal pain regimen) [3, 7]. Previous work has been conducted at large institutions with abundant resources. These results may not have widespread application at institutions with less experience in quality improvement efforts or in contexts where stakeholder reservation exists. The purpose of this study was to assess the value of a modified, minimalistic enhanced recovery protocol in reducing LOS following PSF for adolescent idiopathic scoliosis. In our cohort, minor changes to the timing of mobilization and transition to oral pain medications alone led to a significant decrease in hospital LOS.

Average hospital LOS for AIS patients treated with PSF prior to implementation of ERPs was 5–9 days [2, 13, 14]. In a 2013 retrospective cohort study of 7637 spinal fusion surgical cases, Erickson et al. [15] found that the median hospital LOS for non-neurologically impaired children was 5 days. Recent adoption of ERPs has shown significant reduction in length of hospital stays. Fletcher et al. [16] performed a prospective dual-center study demonstrating a 55% reduction in hospital LOS for patients enrolled in an ERP treatment pathway compared to a traditional discharge pathway (2.2 vs 4.8 days, respectively). Similarly, most patients in the ERP group in our study cohort were discharged on POD 3 (81.2%), whereas most patients in the SRP were discharged on POD 4 (81.8%). Given that there were no differences in perioperative characteristics including the surgeon, number of levels fused, and estimated blood loss, as well as the use of an identical postoperative pain medication regimen in both treatment pathways, the reduction in hospital LOS may be most related to early mobilization. Previously, the advantageous effect of each ERP component was relatively unknown given the simultaneous alteration of multiple variables. The results of this study emphasize the importance of early mobilization as a key factor promoting recovery and decreasing LOS.

Increased pain medication requirement is one concern of early mobilization following PSF for AIS [17]. As such, recent reports have focused on multimodal pain regimens as the foundational component of rapid recovery protocols in AIS [18]. Consistent with most published protocols, Gornitzky et al. [7] implemented a multidisciplinary team-developed analgesic and rehabilitation protocol in AIS patients undergoing PSF to improve pain control and reduce opioid-related complications. They found that a multimodal pain regimen decreased total opioid consumption by POD 0 which was felt to reduce opioid side effects such as opioid-induced pruritis, leading to faster mobilization and recovery. In contrast, the pain medication regimen was identical between the SRP and ERP groups in our study, which reduced the variables confounding assessment of the relative importance of each component of a rapid recovery protocol. The results of the current study suggest that early mobilization may not be a significant contributor to postoperative pain given that there was no difference in hospital narcotic requirements between the SRP and ERP groups. Further, LOS of stay was reduced in our cohort by changing the timing of mobilization without a change to the postoperative pain medications provided to the AIS patients.

Hospital readmission for medical complications, wound dehiscence, surgical site infection, and poor postoperative pain control is a potential concern of early mobilization and decreased hospital length of stay following surgical intervention. The existing enhanced recovery literature does not validate this concern. A study performed by the ERAS Compliance Group examining an international, multicenter registry with over 2300 colorectal patients found implementation of ERPs correlated with both shorter hospital LOS and fewer complications [19]. Gustafsson et al. [20] conducted a retrospective study of 911 colorectal cancer patients and found that patients enrolled in an ERP pathway experienced less postoperative complications and improved 5-year survival rates. Similarly, Fletcher et al. [5] identified no difference in readmissions or wound complications between accelerated discharge pathway and traditional discharge pathway groups for patients with AIS treated with PSF. In our study, there was no difference in complication rates between the ERP and SRP groups. No patients in the ERP group underwent hospital readmission within 30 days, whereas two patients in the SRP group were readmitted. One patient in the SRP group developed superior mesenteric artery syndrome and bowel obstruction 1 month following surgery. There were three complications in the ERP group consisting of one intraoperative pneumothorax, one pleural effusion on POD 1, and one patient with superficial wound dehiscence that was successfully managed in the outpatient setting. These data are consistent with previous literature and confirms that decreasing LOS following AIS is safe and does not increase perioperative risk for AIS patients treated with PSF even at smaller institutions.

The strengths of this study include limited independent variables (single surgeon, single institution, and consecutive patient series). Timing of mobilization and transition to oral pain medication were the only changes between the ERP and SRP groups, which eased resistance to implementation of this postoperative change. At our institution, the positive outcomes from this study led to stakeholder buy-in from anesthesia, nursing, and the other surgeons. Additional protocol changes were then implemented, including an anesthesiology-led multimodal pain regimen, which resulted in further decreases in LOS. Another strength is the applicability of the results to a majority of facilities treating AIS patients, including institutions where reservation to protocol change exists.

This study was limited by its retrospective nature. Patients were not randomized, and data collection was limited to clinical documentation within the electronic medical record. Although our study was significantly powered, the relatively small sample size prevented multivariable analysis, which may have resulted in unaccounted confounding variables. Another limitation of this study was the lack of patient-reported outcomes describing the overall experience and satisfaction with the SRP and ERP pathways. In addition, the study does not account for the culture change among the patient care team as a confounding variable for decreased hospital length of stay. With increased education and established expectations for early mobilization and discontinuation of intravenous pain medication, nursing, and physical therapy teams are likely more amenable and supportive of earlier patient discharge. Future work should focus on isolating each component of an ERP (preoperative counseling, multimodal pain regimen, early mobilization, etc.) to better evaluate each variable’s importance to the care and recovery of AIS patients treated with PSF.

In this study, we instituted a minimally modified postoperative protocol in the setting of stakeholder reservations that reduced patient LOS without increased complications or pain medication requirements. Surgeons who want to initiate an enhanced recovery protocol at institutions with stakeholder reservation or without formal quality improvement teams can use this minimalistic approach as an initial step in the process towards enhanced recovery following surgery for AIS.

## Data Availability

Not applicable.
